# Hemitruncus derecho en la vida adulta: un hallazgo poco usual

**DOI:** 10.47487/apcyccv.v1i3.46

**Published:** 2020-09-30

**Authors:** Rocío Huerta-Robles, Francisco Chávez-Solsol, Zoila Rodríguez-Urteaga, Viviana Nario-Lazo, José Palacios-León, Victor Robles-Velarde

**Affiliations:** 1 Médico residente de Cardiología. Instituto Nacional Cardiovascular - INCOR EsSalud. Lima, Perú. Médico residente de Cardiología Instituto Nacional Cardiovascular - INCOR EsSalud Lima Perú; 2 Servicio de Cardiología Clínica. Instituto Nacional Cardiovascular - INCOR EsSalud. Lima, Perú. Servicio de Cardiología Clínica Instituto Nacional Cardiovascular - INCOR EsSalud Lima Perú; 3 Servicio de Ayuda al Diagnóstico y Tratamiento. Instituto Nacional Cardiovascular - INCOR EsSalud. Lima, Perú. Lima Perú; 4 Unidad de Cardiopatías Congéni- tas del Adulto - Hospital Nacional Edgardo Rebagliati Martins - EsSalud. Lima, Perú. Lima Perú; 5 Servicio de Cirugía de Tórax. Hospital Nacional Guillermo Almenara Irigoyen - EsSalud. Lima, Perú. Lima Perú; 6 Servicio de Cirugía Cardiovascu- lar. Instituto Nacional Cardiovascu- lar - INCOR EsSalud. Lima, Perú. Lima Perú

**Keywords:** Cardiopatías Congénitas, Adulto, Neumonectomía, Heart Defects, Congenital, Adult, Pneumonectomy

## Abstract

El hemitruncus arterioso es una cardiopatía congénita rara caracterizada por el origen anómalo de alguna de las ramas de la arteria pulmonar a partir de la aorta ascendente. En la mayoría de los casos, su diagnóstico es realizado durante la infancia; además, por su elevada morbi-mortalidad es ex- tremadamente inusual su evolución asintomática y supervivencia hasta la etapa adulta. Presentamos el caso de un varón de 30 años, con el antecedente de cierre quirúrgico de persistencia del conducto arterioso en la infancia, que cursó asintomático hasta hace un año, en quien se diagnosticó hemitrun- cus arterioso a partir de episodios recurrentes de hemoptisis.

## Abstract (en)

Hemitruncus arteriosus is a rare congenital heart disease characterized by the abnormal origin of one of the branches of the pulmonary artery from the ascending aorta. In most cases, its diagnosis is made during childhood; in addition, due to its high morbidity and mortality, its asymptomatic evolution and survival to adulthood is extremely unusual. We present the case of a 30-year-old male patient, with a history of surgical closure of patent ductus arteriosus during childhood, who was asymptomatic until a year ago, in whom hemitruncus arteriosus was diagnosed after recurrent episodes of hemoptysis.

El origen anómalo de alguna de las ramas del tronco de la arteria pulmonar a partir de la aorta ascendente se conoce como hemitruncus. Fue descrito por primera vez por Fraentzel en 1868 y tiene una incidencia anual del 0,1% con respecto a todas las cardiopatías congénitas ^1^. La mayor parte de pacientes con hemitruncus arterioso son diagnosticados durante la infancia y, en la mayoría de ellos, es la arteria pulmonar derecha la que se origina anómalamente desde la pared posterior de la aorta ascendente (hemitruncus derecho) ^2^^-^^3^. Con menos frecuencia, es la arteria pulmonar izquierda la que se origina anormalmente de la aorta ascendente (hemitruncus izquierdo) y frecuentemente se asocia con un arco aórtico derecho y tetralogía de Fallot ^2^^-^^4^.

El flujo sanguíneo desde el lado derecho del corazón se dirige al pulmón a través de la arteria pulmonar de origen normal, mientras que la arteria pulmonar de origen anómalo (hemitruncus) transporta sangre oxigenada desde la aorta a los pulmones causando una sobrecarga de volumen y presión, lo que conduce a enfermedad vascular pulmonar progresiva, falla cardíaca y eventualmente la muerte ^4^^-^^5^.

Los niños a quienes se les diagnostica oportunamente esta cardiopatía congénita y se someten a una reparación quirúrgica temprana tienen resultados relativamente mejores. Por otro lado, los niños que no se someten a corrección quirúrgica tienen una tasa de mortalidad a los tres meses de edad y al primer año de vida de 30% y 70%, respectivamente ^6^^-^^7^. Es así que la reparación debe contemplarse dentro de los primeros seis meses de vida para prevenir la enfermedad vascular pulmonar obstructiva severa ^7^.

Se presenta el caso de un paciente de 30 años con esta cardiopatía congénita, quién cursó asintomático hasta la adultez y que fue diagnosticado de forma incidental.

## Descripción del Caso

**Figura 1:**
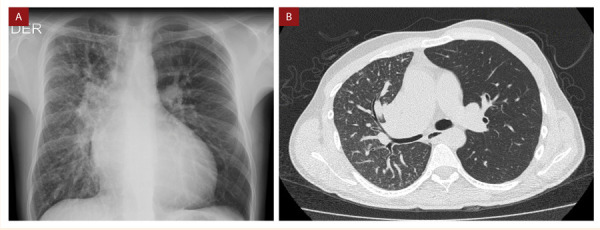
A) Radiografía de tórax: Dilatación de cavidades cardiacas izquierdas y aumento de trama vascular a predominio de pulmón derecho. B) Tomografía de tórax: Reducción de volumen pulmonar derecho con patrón de “vidrio deslustrado” e incremento compensatorio del volumen pulmonar izquierdo.

Paciente varón de 30 años natural y procedente de Lima, con antecedente de cierre quirúrgico de persistencia de conducto arterioso a los 3 años de edad. A partir de entonces, evolucionó asintomático y sin ningún déficit pondo-estatural por lo que los familiares decidieron descontinuar los controles post-operatorios. Sin embargo, en el último año presentó episodios recurrentes de hemoptisis, siendo hospitalizado por un nuevo episodio de mayor severidad con sospecha de tuberculosis pulmonar. Al examen físico, se evidenció un soplo diastólico en foco aórtico III/VI irradiado hacia la región interescapular. El electrocardiograma mostró signos de hipertrofia y sobrecarga sistólica ventricular izquierda. En la radiografía de tórax **(**Figura 1A**)** se evidenció cardiomegalia a expensas de cavidades izquierdas y aumento de la trama vascular a predominio del pulmón derecho.

Con el propósito de descartar una patología infecciosa subyacente, se procedió a realizar una tomografía torácica sin contraste **(**Figura 1B**)** donde se evidenció cardiomegalia, reducción del volumen pulmonar derecho con patrón de “vidrio deslustrado” e incremento compensatorio del volumen pulmonar izquierdo, sin evidencia de hallazgos sugestivos de infección.

**Figura 2:**
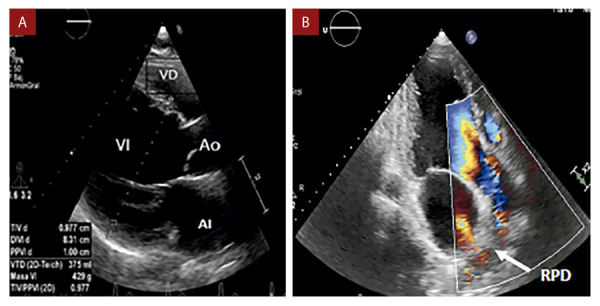
Ecocardiografía transtorácica. **A) Vista para esternal eje largo:** Dilatación severa de cavidades cardiacas izquierdas (diámetro telediastólico de ventrículo izquierdo: 83 mm). **B) Vista apical 3 cámaras:** Se aprecia regurgitación aórtica al colocar doppler color y nacimiento de rama pulmonar derecha a partir de pared posterior de aorta ascendente. RPD: rama pulmonar derecha

En la ecocardiografía transtorácica **(**Figura 2**)** se encontró situs solitus en levocardia, ventrículo izquierdo severamente dilatado con fracción de eyección de 64%, válvula aórtica trivalva displásica con insuficiencia moderada, insuficiencia mitral leve funcional y ventrículo derecho no dilatado con función sistólica conservada (cambio de área fraccional de ventrículo derecho: 35%), evidenciando el origen de la rama pulmonar derecha a partir de la pared posterior de la aorta ascendente.

Ante los hallazgos se realizó una angiotomografía de corazón y grandes vasos **(**Figura 3**y video)** donde se encontró el nacimiento anómalo de la rama pulmonar derecha a partir de la pared posterior de la aorta ascendente con un diámetro de 36 mm (hemitruncus derecho), y nacimiento de la rama pul- monar izquierda de dimensiones conservadas (18 mm) a partir del tronco de la arteria pulmonar y ésta a su vez del ventrículo derecho.

**Figura 3:**
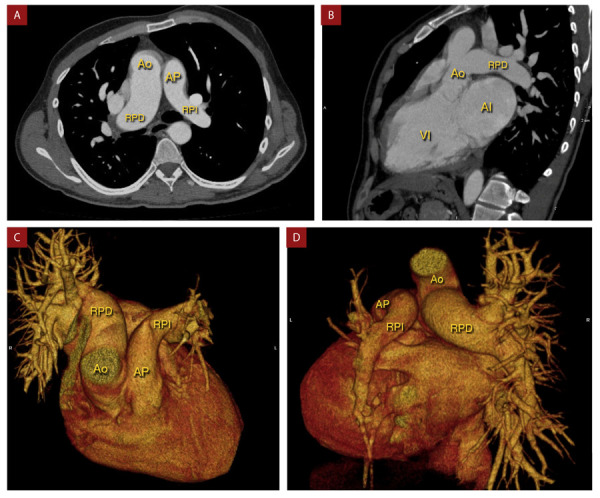
Angiotomografía de corazón y grandes vasos. **A y B)** Nacimiento anómalo de rama pulmonar derecha (RPD) a partir de la pared posterior de la aorta ascendente (Ao). Origen de rama pulmonar izquierda (RPI) a partir de tronco de arteria pulmonar (AP). Obsérvese mayor diámetro de RPD respecto a RPI. **C y D)** Reconstrucción tridimensional de corazón y grandes vasos confirmando los hallazgos.

**Figura 4:**
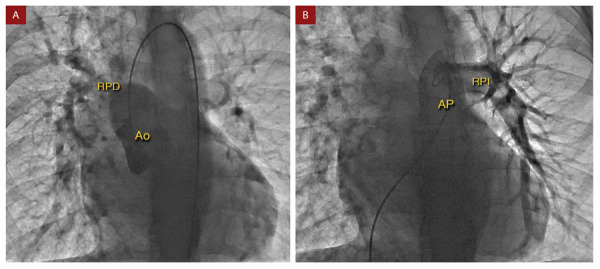
Angiografía. **A) Angiografía aórtica** muestra el origen anómalo de la rama pulmonar derecha (RPD) a partir de la aorta ascendente (Ao). **B) Angiografía pulmonar:** muestra el origen de la rama pulmonar izquierda (RPI) a partir del tronco de la arteria pulmonar (AP).

**Figura 5:**
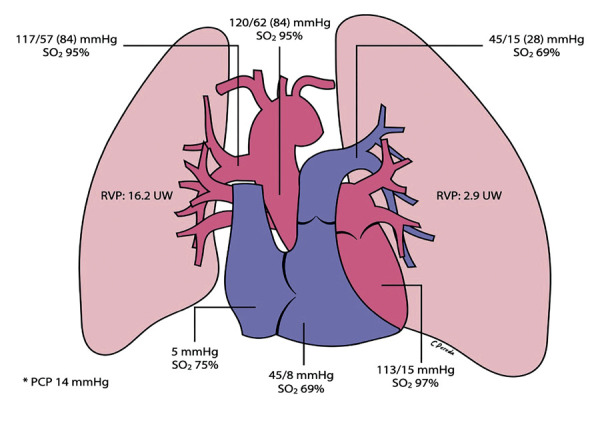
Cateterismo cardíaco y manometría. Hipertensión pulmonar izquierda precapilar (PAPm 28 mmHg; RVP: 2.9 UW) e hipertensión pulmonar derecha con presiones en rango sistémico (PAPm 84 mmHg; RVP: 16.2 UW).

La angiografía pulmonar y aórtica **(**Figura 4**)** confirmó los hallazgos tomográficos, mostrando nacimiento de la rama pulmonar derecha a partir de la aorta ascendente, y nacimiento de rama pulmonar izquierda a partir de tronco de arteria pulmonar. Además, el cateterismo cardíaco evidenció un pulmón izquierdo con hipertensión pulmonar pre-capilar, y el pulmón derecho con medición de presiones equivalentes a la presión arterial sistémica **(**Figura 5**)**. Por las características clínicas y los hallazgos hemodinámicos mencionados, se decidió realizar como tratamiento una neumonectomía derecha.

**Figura 6:**
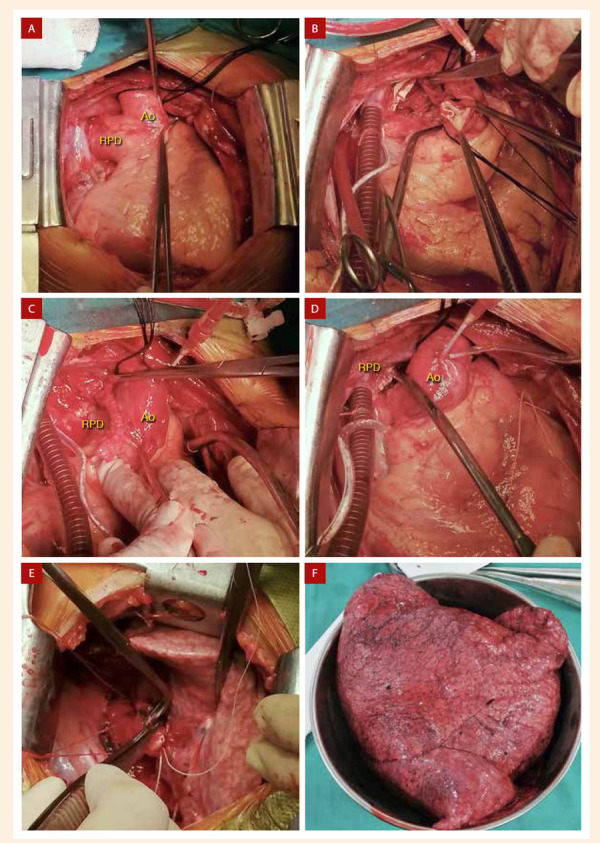
Arterioplastía y neumonectomía derecha. A) Exposición quirúrgica del origen la rama pulmonar derecha (RPD) a partir de la pared posterior de la aorta ascendente (Ao) con un diámetro de 40 mm aproximadamente. B) Exposición de la boca aórtica y pulmonar de la rama pulmonar derecha después de haber sido seccionada. C) Boca aórtica de rama pulmonar derecha (RPD) suturada con polipropileno y reforzada con parche de teflón. D) Boca pulmonar de rama pulmonar derecha (RPD) suturada. E) Neumonectomía derecha. F) Pulmón derecho resecado.

La cirugía se realizó a través de una esternotomía media, canulación arterial y venosa vía femoral, uso de circulación extracorpórea, y parada cardíaca cardiopléjica. Como hallazgos quirúrgicos se encontró el origen anómalo de la rama pulmonar derecha a partir de la pared posterior de la aorta ascendente y dilatación de cavidades cardiacas izquierdas **(**Figura 6A**)**. Se seccionó la arteria pulmonar derecha **(**Figura 6B**)** y se suturó tanto la boca aórtica y pulmonar con polipropileno, reforzando los puntos con parche de teflón **(**Figura 6C**,**6D**)**. Culminada la arterioplastía pulmonar y tras retiro de la circulación extracorpórea, se procedió a realizar una toracotomía lateral derecha, disección del pedículo vascular pulmonar, sección y sutura del componente arterial y venoso, así como del bronquio derecho **(**Figura 6E**)**, y consiguiente neumonectomía derecha **(**Figura 6F**, descripción de la anatomía patológica en suplemento 1).**

Durante el post operatorio se agregó una neumonía intrahospitalaria por lo que se administró tratamiento antibiótico por 14 días. El paciente fue extubado ocho días posteriores a la intervención quirúrgica con éxito y evolucionó favorablemente del proceso infeccioso descrito, siendo dado de alta 17 días después de la intervención quirúrgica. En el control ecocardiográfico post-operatorio a los 45 días, se constató disminución significativa del diámetro telediastólico del ventrículo izquierdo, además de ausencia de nuevos episodios de hemoptisis.

## Discusión

El origen anómalo de una rama arterial pulmonar a partir de la aorta ascendente, con origen de la otra rama a partir del tronco de la arteria pulmonar se conoce como hemitruncus arterioso ^5^. Es importante realizar su diferenciación del tronco arterioso común que sucede cuando el tronco primitivo nunca se divide en aorta y en arteria pulmonar, lo que da origen a un solo tronco arterial que transporta una mezcla de sangre oxigenada y desoxigenada hacia la circulación sistémica, pulmonar y coronaria ^5^.

La incidencia del hemitruncus arterioso es más frecuente en la población pediátrica, siendo extremadamente rara su presentación en la población adulta ^2^^,^^4^. Según lo informado por Haywood *et al.*^4^ sólo se han publicado, hasta la actualidad, diez casos de esta cardiopatía congénita en población adulta y más aun, no existe ningún reporte de su asociación con válvula aórtica displásica como se reporta en este caso.

Se han propuesto varias hipótesis para explicar el origen de esta cardiopatía congénita. La primera sugiere una migración patológica de la arteria pulmonar derecha después de la división normal del tronco de la arteria pulmonar. Otra hipótesis sugiere una división asimétrica del tronco de arteria pulmonar que deja el sexto arco aórtico derecho en el lado incorrecto (aórtico), mientras que una tercera hipótesis se basa en la mal posición izquierda del tabique aortopulmonar ^8^. Hasta la actualidad, se desconoce el mecanismo definitivo.

Las anomalías cardiacas más asociadas a esta cardiopatía son la persistencia de ductus arterioso, la tetralogía de Fallot, el arco aórtico derecho, y los defectos interatriales o interventriculares ^2^, siendo el ductus arterioso persistente la cardiopatía concomitante en nuestro caso.

En el caso presentado, esta cardiopatía congénita se caracteriza por presentar un gasto cardíaco derecho dirigido en su totalidad hacia el pulmón izquierdo (daño por volutrauma); mientras que el gasto cardíaco izquierdo, caracterizado por tener presiones elevadas, se dirige hacia el pulmón derecho a través de la arteria pulmonar derecha que nace patológicamente a partir de la aorta ascendente, condicionando una reacción vasoconstrictora en el lecho vascular pulmonar que se traducirá en hipertensión pulmonar (daño por barotrauma).

El diagnóstico en adultos requiere un alto grado de sospecha clínica, y los estudios imagenológicos no invasivos como la ecocardiografía, tomografía computarizada y la resonancia magnética juegan un rol crucial. Como técnicas invasivas, la angiografía pulmonar y el cateterismo cardíaco ayudan a realizar mediciones hemodinámicas directas y precisas para definir la existencia de hipertensión pulmonar, el tipo, grado de severidad y su potencial reversibilidad ^9^.

En el caso presentado se evidencia hipertensión pulmonar pre-capilar en el pulmón izquierdo e hipertensión pulmonar en el pulmón derecho, pero en este último con presiones en rango sistémico, lo que podría explicar los episodios recurrentes de hemoptisis y el compromiso significativo del parénquima pulmonar derecho observado en la tomografía torácica.

El tratamiento definitivo del hemitruncus en los neonatos y lactantes, edades en las que por lo general se realiza el diagnóstico, consiste en la anastomosis del botón pulmonar anómalo al tronco de la arteria pulmonar y una arterioplastía con parche pericárdico en el sitio del defecto aórtico que dejó la resección del botón vascular ^10^^-^^12^. Sin embargo, este procedimiento quirúrgico implica una alta mortalidad que varía de 2% a 21%, y la tasa de reintervención en el mediano plazo para angioplastía con catéter balón y stent por estenosis de la anastomosis pulmonar es de 2.5% a 36% ^16^. Dentro de los pocos casos reportados de esta cardiopatía en la adultez, el banding de la arteria pulmonar, la neumonectomía y el trasplante pulmonar son las técnicas más frecuentemente utilizadas con buena evolución clínica posterior tanto por mejoría en sintomatología como no necesidad de re-intervenciones posteriores ^13^^-^^15^.

La decisión de neumonectomía derecha en nuestro paciente se basó en los episodios recurrentes de hemoptisis y el gran compromiso del parénquima pulmonar derecho, producto del flujo sanguíneo con presiones en rango sistémico que ha recibido durante sus 30 años de vida, y que ha terminado por producir enfermedad vascular pulmonar (resistencia vascular pulmonar: 16 unidades Woods, ver Figura 5**)**, desestimándose por estos motivos la opción de banding de arteria pulmonar derecha como tratamiento paliativo.

Es difícil esclarecer cuál fue la razón por la cual el caso presentado se mantuvo asintomático por treinta años, teniendo como única manifestación clínica, luego de este periodo, hemoptisis recurrente. Asimismo, la insuficiencia aórtica moderada podría justificar una menor presión de perfusión sistémica hacia el pulmón derecho y con ello, un retraso en la presentación y severidad de las manifestaciones clínicas.
